# Detecting Occlusion Myocardial Infarction with an AI-Powered ECG Model: A Retrospective Cohort Study †

**DOI:** 10.3390/jpm16040174

**Published:** 2026-03-24

**Authors:** Mark B. Hellerman, Cassie Wang, David T. Zhang, Andreas P. Kalogeropoulos, Hal A. Skopicki

**Affiliations:** 1 Division of Cardiology, Department of Medicine, Stony Brook University, Stony Brook, NY 11794, USA; david.zhang@stonybrookmedicine.edu (D.T.Z.); hal.skopicki@stonybrookmedicine.edu (H.A.S.); 2 Department of Medicine, Northwell Northshore University Hospital, Manhasset, NY 11030, USA; cwang21@northwell.edu

**Keywords:** acute coronary syndrome, myocardial infarction, artificial intelligence, occlusion myocardial infarction, ECG

## Abstract

**Background:** Patients with NSTEMI who are found with a totally occluded culprit vessel on coronary angiography are at higher risk of mortality and major adverse cardiac events. Artificial intelligence (AI) models can help identify this subgroup of NSTEMIs. **Objectives**: The purpose of this study was to examine the performance of an AI model in identifying patients with total thrombotic coronary artery occlusion myocardial infarctions (OMIs) using a single 12-lead ECG as input. **Methods**: In this retrospective cohort study, 12-lead ECGs corresponding to patients with suspected OMI were analyzed by an AI model. Confirmation of OMI was based on angiographic evidence of acute culprit coronary artery stenosis. **Results**: Over a one-year period, emergency physicians at our hospital identified 474 patients with suspected OMI, of whom 88 met STEMI criteria. Out of the 142 angiographically confirmed OMIs, the AI model correctly identified 115 (81%) with high confidence, corresponding to an accuracy of 89.4%, sensitivity of 90.0%, specificity of 93.2%, positive predictive value (PPV) of 84.6%, and negative predictive value (NPV) of 91.4%. Out of the 74 angiographically confirmed OMIs that did not meet STEMI criteria, the AI model correctly identified 49 (66%) with high confidence, corresponding to an accuracy of 87.9%, sensitivity of 66.2%, specificity of 93.4%, PPV of 72.1%, and NPV of 91.5%. Out of the 68 angiographically confirmed OMIs that met STEMI criteria, the AI model correctly identified 66 (97%) with high confidence, corresponding to an accuracy of 95.5%, sensitivity of 97.1%, specificity of 90.0%, PPV of 97.1%, and NPV of 90.0%. **Conclusions:** The AI model examined in this study outperformed the STEMI criteria for the identification of OMI with respect to accuracy, sensitivity, specificity, PPV, and NPV and accurately identified a significant portion of NSTEMIs found to have total thrombotic coronary artery occlusion.

## 1. Introduction

Historically, the triage of patients with acute coronary syndrome (ACS) has been dichotomized on the basis of millimeter changes in the amplitude of the ST-segment on the electrocardiogram (ECG) [1]. Current guidelines support emergent coronary angiography for patients presenting with ST-segment elevation myocardial infarctions (STEMIs) while permitting delayed coronary angiography for patients presenting with non-ST-segment elevation myocardial infarctions (NSTEMIs) [1]. Although the ST-segment elevation observed in STEMIs is thought to manifest from injury currents generated by total thrombotic occlusion of a coronary artery, ST elevation is not observed in 25–35% NSTEMIs with underlying total thrombotic occlusion at the time of cardiac catheterization (STEMI-negative occlusion myocardial infarctions, or STE (−) OMIs) [2]. In recent years, the OMI paradigm has been proposed as a physiologically grounded alternative to the STEMI/NSTEMI classification, emphasizing the identification of acute coronary artery occlusion irrespective of ST-segment elevation criteria [2,3]. Under this framework, OMI refers to acute coronary occlusion or near-occlusion requiring urgent reperfusion, whereas non-occlusion MI (NOMI) encompasses infarctions without an acutely occluded culprit artery. This distinction differs from myocardial infarction due to oxygen supply–demand mismatch, which may occur without acute plaque rupture or occlusive thrombus. Mortality rates in this subgroup of STE (−) OMIs have been reported to be at least twice as high as rates reported in NOMIs [4].

The reliable identification of STE (−) OMIs is clinically important, as this is a high-risk subgroup of NSTEMIs that may benefit from expedited coronary angiography [2]. Several ECG criteria beyond those defined for STEMI in the 4th Universal Definition of MI have demonstrated high sensitivity and specificity for identifying this subgroup [5,6,7,8,9,10,11,12]. Recently, the American College of Cardiology (ACC) categorized five of these criteria as “STEMI equivalents”, including (1) posterior STEMI, (2) and (3) left bundle branch block meeting Sgarbossa or Smith-modified Sgarbossa criteria, (4) De Winter Sign, and (5) hyperacute T waves [13]. While an expansion of the list of “STEMI equivalents” can be expected to expedite angiography for previously missed OMIs, inter-reader agreement remains poor for both the original and expanded STEMI criteria [14,15,16].

Additional criteria for identifying STE (−) OMIs have been described and validated; however, the increased time required to master these criteria limits their clinical impact. Thus, deep learning artificial intelligence (AI) models have been considered for their potential to make acute occlusion myocardial infarction (OMI) pattern recognition more accessible, efficient, and reproducible [17,18,19]. An explainable AI model by PMCardio, the “Queen of Hearts” (QoH) model, was recently trained to identify several ECG patterns predictive of OMI using a single 12-lead ECG as input [20,21].

This retrospective cohort study aimed to evaluate the performance of the QoH AI model in detecting OMIs in patients who presented to a regional cardiac catheterization referral center with suspected OMI.

## 2. Materials and Methods

### 2.1. Data Sources and Processing

The Stony Brook University Hospital (SBUH) Emergency Department has established a dedicated pathway—termed “OMI Alert”—to activate urgent cardiology consultation for consideration of early coronary angiography for patients presenting with suspected OMIs. OMI Alert activation is initiated by the treating emergency physician based on clinical judgment, incorporating (1) symptoms suggestive of acute coronary syndrome (e.g., chest discomfort, dyspnea, and epigastric pain) and (2) ECG findings that are concerning for acute coronary occlusion. These ECG findings include fulfillment of STEMI criteria, ACC-recognized STEMI equivalents, or other ECG patterns suggestive of OMI at the physician’s discretion. Activation is not based on a rigid algorithm but reflects real-world clinical decision-making within a structured triage pathway.

**Figure 1:**
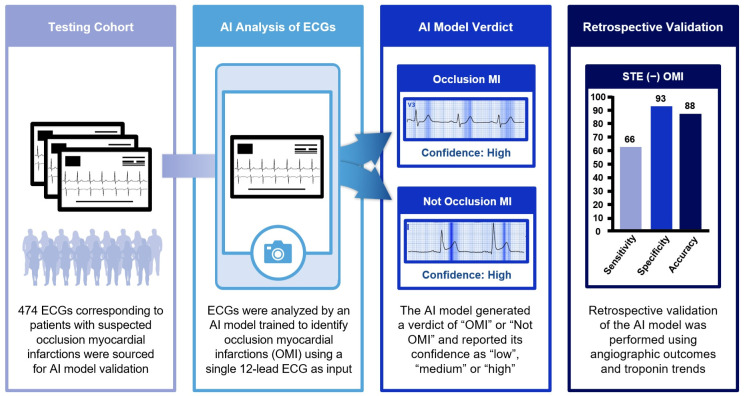
Overview of study design: Identification of occlusion myocardial infarction using an AI-powered ECG model.

**Figure 2:**
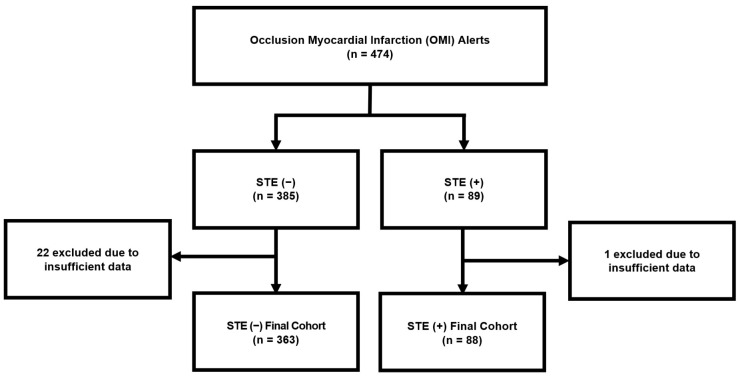
Study inclusion flow diagram.

ECGs from 451 consecutive patients presenting with suspected STE (−) and STE (+) OMIs to SBUH between July 2022 and July 2023 were analyzed by the QoH AI model (Figure 1 and Figure 2). The median age was 68 years, of whom 66% were men. Of these patients, 23 expired before sufficient data was collected to determine if ECG findings were attributable to OMI—these patients were excluded from the testing cohort. This retrospective study was approved by the Institutional Review Board of Stony Brook University Hospital (code 2023-00367, approved on 30 December 2023). Informed consent was waived due to the retrospective nature of the study.

### 2.2. Definitions

The presence of STEMI was assessed based on the 4th Universal Definition of Myocardial Infarction and included new ST-elevation (STE) ≥1 mm in two contiguous leads other than leads V2–V3 (where STE ≥2 mm in men ≥40 years, ≥2.5 mm in men <40 years, and ≥1.5 mm in women). Time from the first ECG to intervention was recorded for all cases if the patient underwent coronary angiography. Outcomes were adjudicated as OMI or not OMI by clinically validated angiographic outcome data. “Not OMI’’ encompasses patients who either do not have acute MI or have acute non-occlusion MI (Non-OMI, or NOMI) with either no culprit vessel identified angiographically or where the identified culprit vessel does not require immediate revascularization.

### 2.3. Algorithm

**Figure 3:**
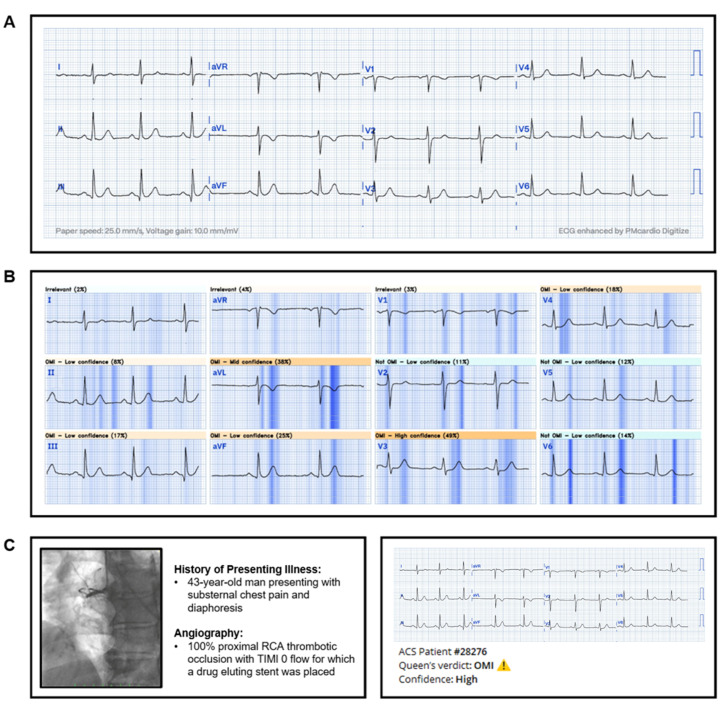
Example of an AI model verdict for an STE (−) OMI patient (**A**). Example of the visual explanation the AI model produced to provide insight into which portions of the ECG contributed most to its final verdict (**B**). Clinical vignette of the patients (**C**, **left**) and corresponding AI model output (**C**, **right**).

**Figure 4:**
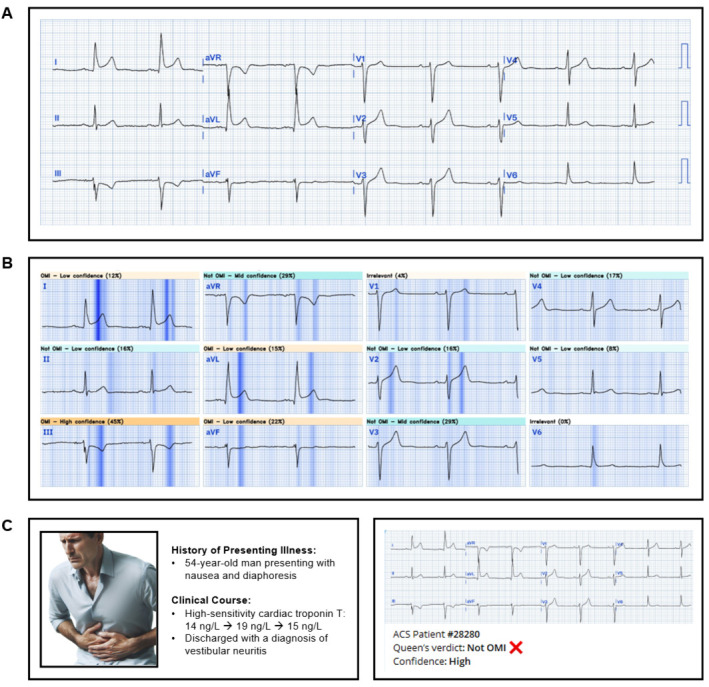
Example of an AI model verdict for a false-positive STEMI patient. This is an example of an ECG meeting STEMI criteria (**A**) from a patient who was ruled out for acute coronary syndrome by serial troponins. Example of the visual explanation the AI model produced to provide insight into which portions of the ECG contributed most to its final verdict (**B**). Clinical vignette of the patient (**C**, **left**) and corresponding AI model output (**C**, **right**).

The QoH AI model provides a binary prediction of OMI or not OMI based on a single 12-lead ECG input. Along with a verdict of OMI or not OMI, the model categorizes its confidence level into low, medium, or high (Figure 3 and Figure 4) according to predefined probability thresholds established during model development by the vendor. These thresholds were fixed and not modified for this validation study. For analysis, we evaluated model performance using progressively inclusive thresholds (high only; medium + high; low + medium + high).

### 2.4. Primary Outcome

The primary outcome was the AI model’s ability to identify patients with angiographically confirmed OMI using a single standard 12-lead ECG. The primary definition of OMI was modeled from previous studies and consisted of clinical symptoms and a troponin elevation consistent with the 4th Universal Definition of MI and angiographic evidence of acute culprit coronary stenosis with either (a) thrombolysis in myocardial infarction (TIMI) flow grade of 0–1; or (b) TIMI flow grade of 2–3 with emergent or urgent percutaneous revascularization. Independent clinical reviewers adjudicated the angiographic data of all patients included in the dataset.

### 2.5. Statistical Analysis

The performance of the AI model was evaluated using accuracy, sensitivity, specificity, positive predictive value (PPV), and negative predictive value (NPV). Accuracy was defined as:

$$\mathrm{Accuracy}=\frac{TP+TN}{TP+TN+FP+FN}$$

Sensitivity was prioritized to assess the model’s ability to detect angiographically confirmed OMIs and minimize missed occlusions. Specificity was included to evaluate the potential reduction in unnecessary catheterization laboratory activation. Predictive values were calculated to enhance clinical interpretability within this suspected OMI population; however, these metrics are prevalence-dependent and may vary in other settings. Performance was further stratified by the AI model’s confidence in its verdict—categorized as either high, medium, or low confidence. Statistical analysis was performed using SPSS Statistics Software V31.

## 3. Results

The OMI Alert study cohort included 363 ECGs from patients presenting with suspected STE (−) OMIs, of whom 214 (46%) had a rise and fall in troponin value recorded higher than the 99th percentile. All patients had symptoms of either chest discomfort, dyspnea, or epigastric pain but did not have ECG findings consistent with STEMI criteria as defined by the 4th Universal Definition of Myocardial Infarction. In total, 22 patients were excluded due to death prior to the collection of sufficient clinical data to determine if OMI had occurred. Angiographic evidence of acute thrombotic occlusion was found in 18% (67/363) of the total cohort and in 31% (66/214) of patients with a rise in troponin greater than the 99th percentile. Out of the 67 STE (−) OMIs, 58 received PCI, 8 were referred for CABG, and 1 was discharged with a diagnosis of MINOCA.

### 3.1. Model Performance in STEMI-Negative Cohort

When a restrictive OMI confidence threshold was enforced (restricting verdicts to those with a high level of confidence), the AI model correctly identified 66% (49/74) of true OMIs, missed 34% (25/74) of true OMIs, and over-called 6.6% (19/289) of true NOMIs, corresponding to an accuracy of 87.9%, sensitivity of 66.2%, specificity of 93.4%, PPV of 72.1%, and NPV of 91.5% (Table 1).

When a broader OMI confidence threshold was permitted (restricting verdicts to those with a medium or high level of confidence), the AI model correctly identified 82% (61/74) of true OMIs, missed 17.5% (13/74) of true OMIs, and over-called 10.7% (31/289) of true NOMIs, corresponding to an accuracy of 87.9%, sensitivity of 82.4%, specificity of 89.3%, PPV of 66.3%, and NPV of 95.2% (Table 1).

When the broadest OMI confidence threshold was permitted (encompassing verdicts with a low, medium, or high level of confidence), the AI model correctly identified 90.5% (67/74) of true OMIs, missed 34% (25/74) of true OMIs, and over-called 6.6% (19/289) of true NOMIs, corresponding to an accuracy of 84.8%, sensitivity of 90.5%, specificity of 83.4%, PPV of 58.3%, and NPV of 97.2% (Table 1).

Median ECG-to-balloon (E2B) time for OMIs in this study was 61 min, with a median time of 48 min for STE (+) OMIs and a median time of 104 min for STE (−) OMIs (Table 1). Among STE (+) OMIs, the median E2B time was 41 min for men and 53 min for women. Among STE (−) OMIs, the median E2B time was 92 min for men and 155 min for women. E2B time was greater than 2 h for 48% (31/64) of the STE (−) OMIs, with a median E2B time of 458 min. E2B time was achieved in less than 2 h (median time 62 min) in 51% (33/64) of the STE (−) OMIs. Of the 31 STE (−) OMIs with E2B greater than 2 h, the QoH model identified 22 (70%) with high confidence. Of the 19 false-positive STE (−) OMIs identified using the restrictive threshold, 10 (53%) underwent coronary angiography within 1 month of the index hospitalization.

### 3.2. Model Performance in STEMI-Positive Cohort

In total, 88 ECGs that fulfilled STEMI criteria were analyzed by the QoH AI model in this study. When a restrictive OMI confidence threshold was enforced (restricting verdicts to those with a high level of confidence), the QoH model correctly identified 97% (66/68) of true STE (+) OMIs, missed 3% (2/68) of true STE (+) OMIs, and over-called 10% (2/20) of true STE (+) NOMIs, corresponding to an accuracy of 95.5%, sensitivity of 97.1%, specificity of 90.0%, PPV of 97.1%, and NPV of 90.0% (Table 1).

When a broader OMI confidence threshold was permitted (restricting verdicts to those with a medium or high level of confidence), the QoH model correctly identified 100% (68/68) of true STE (+) OMIs, missed 0% (0/68) of true STE (+) OMIs, and over-called 20% (4/20) of true STE (+) NOMIs, corresponding to an accuracy of 95.5%, sensitivity of 100%, specificity of 80.0%, PPV of 94.4%, and NPV of 100% (Table 1).

When the broadest OMI confidence threshold was permitted (encompassing verdicts with a low, medium, or high level of confidence), the AI model correctly identified 100% (68/68) of true STE (+) OMIs, missed 0% (0/68) of true STE (+) OMIs, and over-called 20% (4/20) of true STE (+) NOMIs, corresponding to an accuracy of 95.5%, sensitivity of 100%, specificity of 80.0%, PPV of 94.4%, and NPV of 100% (Table 1).

In our cohort, 23% (20/88) of STEMIs were false positives—with 70% (14/20) having been ruled out by negative troponin trends. Coronary angiography revealed nonobstructive disease in 30% (6/20) of these cases. Of the 16 false-positive STEMIs that the AI model correctly identified, ST elevation was attributed to left ventricular hypertrophy in six cases, benign early repolarization in five cases, pericarditis in four cases, and left ventricular aneurysm in one case.

Two angiographically confirmed STE (+) OMIs were not identified at the restrictive (high-confidence) threshold. Both cases demonstrated borderline ST-segment elevation amplitudes with competing repolarization abnormalities, resulting in lower model confidence. Importantly, because this study was retrospective, AI output did not influence clinical decision-making, and both patients underwent timely coronary angiography based on standard STEMI activation protocols.

### 3.3. Model Performance Among All OMIs

In total, 142 OMIs were angiographically confirmed in this study cohort, of which 74 (52%) did not meet STEMI criteria. The QoH model correctly identified 115 (80.9%) of these cases with a high level of confidence, corresponding to an accuracy of 89.4%, sensitivity of 90.0%, specificity of 93.2%, PPV of 84.6%, and NPV of 91.4% (Table 1).

### 3.4. Model Performance by Culprit Lesion

Of the 142 OMIs identified in this study, angiography revealed that most of the culprit lesions were located in the left anterior descending coronary artery (LAD, 50%), followed by the right coronary artery (RCA, 33%), and then the left circumflex coronary artery (LCX, 11%) (Table 2). When a restrictive confidence threshold was enforced (restricting verdicts to those with a high level of confidence), the most common missed culprit lesions were LAD and LCX—each accounting for three of seven missed OMIs, followed by the ramus intermedius coronary artery (RI)—accounting for one of seven missed OMIs. No RCA lesions were missed in this validation study.

## 4. Discussion

This retrospective validation study demonstrates that the QoH AI model can identify angiographically confirmed occlusion myocardial infarction in a real-world suspected OMI cohort using a single 12-lead ECG. When stratified by confidence threshold, the model demonstrated favorable sensitivity and specificity compared with STEMI criteria alone. While these findings are encouraging, they should be interpreted as an incremental step in the validation pathway of AI-assisted OMI detection rather than as definitive evidence of clinical outcome benefit.

Since the publication of the 1st Universal Definition of MI in 2000, the criteria for STEMI have undergone several adaptations in view of optimizing its predictive value for identifying acute thrombotic coronary artery occlusion [13,22,23,24]. Despite the relatively simple and well-defined criteria outlined in the 3rd and 4th Universal Definitions of MI, previous studies found poor inter-reader agreement among physicians, with sensitivity and specificity for OMI identification between 65–70% and 72–86%, respectively—resulting in an unnecessary cardiac catheterization lab activation rate of 36% [25,26,27]. Contemporary efforts to refine AMI classification, including the DIFOCCULT-3 trial, highlight the importance of distinguishing occlusion-driven infarction from supply–demand mismatch infarction in both diagnostic and therapeutic decision-making [28].

There is an ongoing effort to establish additional ECG criteria to identify acute thrombotic lesions not captured by STEMI—which currently account for 25–35% of NSTEMIs [2]. Five such criteria were recently recognized by the ACC as “STEMI equivalents,” and several others have been validated in smaller studies [13]. Although among self-identified ECG experts, these criteria demonstrate high sensitivity and specificity for OMI detection, among less experienced readers, their clinical impact has been limited and suffers from poor inter-reader agreement [14,27].

In this study, we found that 80% of the suspected STE (−) OMIs identified by emergency physicians had no laboratory or angiographic evidence of OMI. The QoH AI model appropriately identified 78% (227/289) of this cohort as false-positive activations with a medium–high level of confidence while capturing 61 true STE (−) OMIs. Within our cohort of STE (+) OMIs, the QoH model appropriately identified all OMIs with a medium–high level of confidence, capturing only four false-positive OMIs. In the future, we imagine that AI models such as this one may not only be useful in augmenting clinical decision making for the triage of patients presenting with suspected OMI but may also serve as a helpful educational tool for trainees and physicians trying to improve their OMI recognition skills.

Among STE (−) OMIs in our cohort, 48% had an E2B time greater than 2 h, with a median E2B of 458 min (Table 2). While these triage intervals are consistent with those reported by institutions with similar triage pathways for early OMI identification, we suspect that E2B intervals might be even longer at hospitals that lack such pathways and that AI models demonstrate potential to assist in triage [27,29]. Notably, among women with STE (−) OMIs in our cohort, median E2B time was nearly 1.7-fold greater (155 min vs. 92 min) than for their STE (−) OMI male counterparts. Several studies have established that women who present with NSTEMI are at higher risk for adverse outcomes compared to men, with a 5-year risk of death being almost 1.5-fold higher (42% for women vs. 29% for men) [30]. The reason for this disparity is likely multifactorial; however, one important component may be a greater time delay to reperfusion among the subset of women presenting with NSTEMs who have a total thrombotic coronary artery occlusion. AI models such as this one, which are blinded to patient sex, may help triage algorithms minimize this disparity.

The contribution of this study is incremental but clinically relevant. Prior investigations have demonstrated that machine learning models can outperform traditional STEMI criteria in detecting occlusion-related infarction. A previous study described a machine learning model that outperformed clinicians in the detection of STE (−) OMIs [18]. Clinical adoption of the model described in that study may be limited, however, as it required a specific ECG file format for input. The QoH AI model is more versatile in this regard, as it utilizes CE-certified PMcardio ECG digitization technology to analyze ECG tracings from a broad array of image formats, including those captured by mobile phone cameras and computer screenshots. To our knowledge, this is the first study validating the QoH AI model in a subset of patients with suspected OMI, stratifying performance by model confidence. Our study extends this literature by validating an explainable AI model within a real-world OMI triage pathway, stratifying performance by model confidence and examining diagnostic performance separately in STE (+) and STE (–) cohorts. Nonetheless, prospective multicenter validation will be required before widespread implementation can be recommended.

Historically, the accurate diagnosis of ACS represented a major challenge for deep learning AI systems; however, this study contributes to a growing body of literature demonstrating a promising future for their ability to augment clinical decision making [17,31,32]. The accurate and reproducible nature of these models provides a foundation for future prospective studies to determine whether the earlier angiography of STE (−) OMIs improves mortality. We also believe that the explainable nature of these models may be leveraged in the future for educational purposes to help train clinicians to better recognize OMI ECG patterns.

Importantly, this study evaluates diagnostic performance rather than clinical outcomes. Although the earlier identification of occlusion myocardial infarction is biologically plausible to improve outcomes, this study does not demonstrate reductions in mortality, reinfarction, heart failure, or major adverse cardiovascular events. Prospective outcome-driven trials are necessary to determine whether AI-guided triage strategies translate into improved patient-centered outcomes.

### Limitations

There are several limitations to our study. First, this was a retrospective, single-center study conducted at an institution with a dedicated OMI Alert pathway. Patient selection, ECG acquisition systems, and triage workflows may differ at other centers, potentially affecting model performance and limiting external generalizability. Second, the study population consisted of patients already suspected of having OMI, introducing spectrum bias and limiting extrapolation to unselected emergency department populations. Predictive values reported here are therefore context-dependent. Third, although diagnostic performance metrics were favorable, this study did not assess clinical outcomes. Whether AI-assisted identification of STE (–) OMI leads to improved mortality or reduced major adverse cardiac events remains unknown. Fourth, subgroup analyses, including culprit lesion-specific performance, were limited by modest sample sizes and should be interpreted cautiously. Fifth, one important subgroup that was excluded from our study was out-of-hospital cardiac arrest (OHCA) patients who died prior to cardiac catheterization. Thus, the findings of our study should not be applied to patients presenting with OHCA. Recent studies have found that 17–43% of OHCA patients without STEMI on initial ECG after the return of spontaneous circulation (ROSC) are found with acute unstable lesions at the time of angiography [33,34]. The abnormal metabolic milieu present within the first 15 min after ROSC is thought to contribute to the diagnostic challenge of appreciating ECG changes secondary to ACS [35]. In the future, it would be helpful to examine the performance of this model on post-ROSC ECGs to determine if it carries the potential to improve outcomes in this high-risk patient population. Finally, although we found that women presenting with STE (−) OMI in this study had a prolonged triage time to angiography compared to men, we were unable to determine the etiology of this discrepancy or address possible confounders, given that this study was not designed to evaluate causal mechanisms or adjust for potential confounders. Many studies have found that atypical symptoms of ACS (such as back pain, nausea, dyspnea, acute fatigue, and lack of pain) are more common in women compared to men [30]. We hypothesize that this may have contributed to the discrepancy in time to angiography observed in women presenting with STE (−) OMI in this study. In the absence of typical anginal symptoms, clinicians may have been inclined to wait for a troponin trend before activating the catheterization lab in some of these scenarios. In future studies of STE (−) OMI, it would be helpful to determine if atypical anginal symptoms at presentation correlate with a propensity to wait for a troponin trend prior to angiography.

## 5. Conclusions

In this retrospective single-center cohort of patients with suspected occlusion myocardial infarction, the QoH AI model demonstrated favorable sensitivity and specificity compared with the STEMI criteria for the identification of angiographically confirmed OMI. These findings support further prospective, multicenter evaluation. Whether AI-assisted detection of STE (–) OMI improves clinical outcomes remains to be determined.

## Figures and Tables

**Table 1:** AI model performance stratified by level of confidence.

	AI Model Level of Confidence in OMI Verdict		
	High	Medium–High	Low–High
STE (−) Cohort (*n* = 363)			
True Positive	49	61	67
True Negative	270	258	241
False Positive	19	31	48
False Negative	25	13	7
Accuracy	87.9%	87.9%	84.8%
Sensitivity	66.2%	82.4%	90.5%
Specificity	93.4%	89.3%	83.4%
Positive Predictive Value	72.1%	66.3%	58.3%
Negative Predictive Value	91.5%	95.2%	97.2%
STE (+) Cohort (*n* = 88)			
True Positive	66	68	68
True Negative	18	16	16
False Positive	2	4	4
False Negative	2	0	0
Accuracy	95.5%	95.5%	95.5%
Sensitivity	97.1%	100%	100%
Specificity	90.0%	80.0%	80.0%
Positive Predictive Value	97.1%	94.4%	94.4%
Negative Predictive Value	90.0%	100%	100%
Total Cohort (*n* = 451)			
True Positive	115	129	135
True Negative	288	274	257
False Positive	21	35	52
False Negative	27	13	7
Accuracy	89.4%	89.4%	86.9%
Sensitivity	90.0%	90.9%	95.1%
Specificity	93.2%	88.7%	83.2%
Positive Predictive Value	84.6%	78.7%	72.2%
Negative Predictive Value	91.4%	95.5%	97.4%

AI: artificial intelligence; OMI: occlusivee myocardial infarction; STE: ST-eleveation.

**Table 2:** Angiographic outcomes of confirmed OMIs.

	Confirmed Occlusion Myocardial Infarctions (OMIs)		
	All (*n* = 142)	STE (−) (*n* = 74)	STE (+) (*n* = 68)
Culprit lesion (%)			
LM	5% (7)	3% (3)	6% (4)
LAD	50% (72)	48% (35)	55% (37)
RCA	33% (47)	38% (28)	28% (19)
LCx	11% (16)	11% (8)	12% (8)
Culprit lesion (%) among OMIs missed by AI model (*n* = 7)			
LM	0% (0)	0% (0)	N/A
LAD	43% (3)	43% (3)	N/A
RCA	0% (0)	0% (0)	N/A
LCx	43% (3)	43% (3)	N/A
RI	14% (1)	14% (1)	N/A
ECG to Balloon (E2B) Time			
Total Cohort	61 min	104 min	48 min
Men	59 min	92 min	41 min
Women	106 min	155 min	53 min
E2B Time > 120 min	*n* = 36 (25%)	*n* = 36 (48%)	*n* = 0
Median Time (min)	458 min	458 min	N/A

E2B = ECG to balloon; LAD = left anterior descending; LCx = left circumflex; LM = left main; RCA = right coronary artery; RI = ramus intermedius; STE: ST-elevation; AI: artificial intelligence; ECG: electrocardiogram; N/A: not applicable.

## Data Availability

The original contributions presented in this study are included in the article. Further inquiries can be directed to the corresponding authors.

## Abbreviations

The following abbreviations are used in this manuscript:

ACC	American College of Cardiology
ACS	Acute coronary syndrome
AI	Artificial intelligence
E2B	Electrocardiogram-to-balloon
ECG	Electrocardiogram
LAD	Left anterior descending coronary artery
LCX	Left circumflex coronary artery
LM	Left main coronary artery
NOMI	Non-occlusion myocardial infarction
NPV	Negative predictive value
NSTEMI	Non-ST-segment elevation myocardial infarction
OMI	Occlusion myocardial infarction
PPV	Positive predictive value
QoH	Queen of Hearts
RCA	Right coronary artery
RI	Ramus intermedius coronary artery
ROSC	Return of spontaneous circulation
SBUH	Stony Brook University Hospital
STE (−) OMI	Occlusion myocardial infarction not meeting STEMI criteria
STE (+) OMI	Occlusion myocardial infarction meeting STEMI criteria
STEMI	ST-segment elevation myocardial infarctions
